# Isolation of Aggressive Behavior Mutants in *Drosophila* Using a Screen for Wing Damage

**DOI:** 10.1534/genetics.117.300292

**Published:** 2017-11-06

**Authors:** Shaun M. Davis, Amanda L. Thomas, Lingzhi Liu, Ian M. Campbell, Herman A. Dierick

**Affiliations:** * Department of Molecular and Human Genetics, Baylor College of Medicine, Houston, Texas 77030; † Department of Neuroscience, Baylor College of Medicine, Houston, Texas 77030; ‡ Program in Developmental Biology, Baylor College of Medicine, Houston, Texas 77030

**Keywords:** *Drosophila melanogaster*, *Shaker*, aggression, copulation, damage, EMS mutagenesis, flight, flight assay, whole-genome sequencing

## Abstract

Aggression is a complex social behavior that is widespread in nature. To date, only a limited number of genes that affect aggression have been identified, in large part because the complexity of the phenotype makes screening difficult and time-consuming regardless of the species that is studied. We discovered that aggressive group-housed *Drosophila melanogaster* males inflict damage on each other’s wings, and show that wing damage negatively affects their ability to fly and mate. Using this wing-damage phenotype, we screened males from ∼1400 chemically mutagenized strains and found ∼40 mutant strains with substantial wing damage. Five of these mutants also had increased aggressive behavior. To identify the causal mutation in one of our top aggressive strains, we used whole-genome sequencing and genomic duplication rescue strategies. We identified a novel mutation in the voltage-gated potassium channel *Shaker* (*Sh*) and show that a nearby previously identified *Sh* mutation also results in increased aggression. This simple screen can be used to dissect the molecular mechanisms underlying aggression.

## Abstract (precis)

Genetic screens have been extremely fruitful to identify mechanistic components in a broad range of biological processes. Aggressive behavior has largely...

AGGRESSIVE behavior is pervasive throughout the animal kingdom. While beneficial to help animals compete for limited resources such as food and mates, aggressive encounters can also lead to physical damage and in some instances even death (Watts *et al.* 2006; Umbers *et al.* 2012; Georgiev *et al.* 2013). *Drosophila melanogaster* has recently emerged as a powerful model organism to study this trait because of the numerous genetic and neurobiological tools available in this species (Jones 2009; Venken *et al.* 2011; del Valle Rodriguez *et al.* 2012), in addition to their distinctive aggressive behaviors first described in detail more than half a century ago (Jacobs 1960). In the last 15, years a range of studies have explored some of the underlying mechanisms regulating aggression by examining the role of sensory systems (Wang and Anderson 2010; Liu *et al.* 2011; Wang *et al.* 2011; Andrews *et al.* 2014), sex-determining pathways (Lee and Hall 2000; Vrontou *et al.* 2006; Fernandez *et al.* 2010; Hoopfer *et al.* 2015; Koganezawa *et al.* 2016), neurotransmitter systems (Baier *et al.* 2002; Certel *et al.* 2007; Dierick and Greenspan 2007; Hoyer *et al.* 2008; Zhou *et al.* 2008; Alekseyenko *et al.* 2010; Andrews *et al.* 2014), and neurosecretory cells and/or the neuropeptides they produce (Asahina *et al.* 2014; Davis *et al.* 2014). These investigations have provided great insight into fly aggression, but were driven largely by specific hypotheses involving known or expected factors regulating various aspects of this behavior. Recently, an unbiased screen was performed to identify small neuronal populations that can trigger a strong aggression response when acutely activated with a thermosensitive TrpA1 (Transient receptor potential cation channel A1) channel (Hoopfer *et al.* 2015). Conditional activation of a subset of P1 neurons induced immediate courtship behavior and long-lasting aggressive behavior in male flies. More recently, this circuit was further dissected and found to be a double-negative regulation involving both Doublesex (Dsx) and Fruitless (Fru) (Zampolini *et al.* 2012) neurons in the P1 cluster, where the Dsx neurons regulate aggression and the Fru neurons control courtship (Koganezawa *et al.* 2016).

Forward genetic chemical mutagenesis screens have been a powerful methodology to unravel the molecular components of many biological processes, including behavior. This unbiased approach led to the discovery of the first mutants in circadian rhythm (Konopka and Benzer 1971), courtship (von Schilcher 1977), learning and memory (Dudai *et al.* 1976; Quinn *et al.* 1979), and, more recently, identified novel components involved in ethanol intoxication (Singh and Heberlein 2000) and sleep (Cirelli *et al.* 2005; Stavropoulos and Young 2011). A similar approach to identify novel mutant alleles in genes that regulate aggression has never been reported in *Drosophila* or any other species because of the complex nature of the behavioral phenotype. Direct observation of altered aggression is time-consuming, which has so far made high-throughput screens challenging. To circumvent this obstacle, we explored whether an indirect and easier phenotype could be scored as a read-out of aggressive behavior. In most species, aggression can cause physical damage as a result of intensely aggressive encounters. Many examples exist within the animal kingdom of lasting scars to the head, body, or limbs after ferocious physical combat (Umbers *et al.* 2012; Georgiev *et al.* 2013), but in flies such evidence has been lacking.

Here, we show that flies also exhibit lasting marks of physical damage to their wings as a result of fighting. Not surprisingly, this damage also results in a negative consequence to the males’ flight and mating abilities. We used this easy-to-score phenotype to perform the first forward chemical mutagenesis screen to isolate mutants with increased aggression. Of the roughly 1400 lines that we screened, 41 had significant wing damage and, of those, five lines showed increased aggressive behavior. Using whole-genome sequencing and duplication mapping strategies, we identified the causal variant in one of the top aggressive lines. This variant alters an evolutionarily conserved residue in the voltage-gated potassium channel, *Shaker*, suggesting that regulation of neuronal activity can profoundly affect a complex behavior such as aggression. Together, these results show that aggression-induced wing damage can be used to successfully screen for novel components involved in the regulation of aggressive behavior in *Drosophila*.

## Materials and Methods

### Fly stocks and rearing conditions

The following fly strains were obtained from the Bloomington *Drosophila* Stock Center: *Sh^mns^* (BL24149), *Sh^5^* (BL111), *Sh^14^*, and the duplication stocks listed in Supplemental Material, Table S1 in File S1. The C(1)DX,*y w f* strain was a kind gift from Richard Kelley (Baylor College of Medicine, Houston, TX). The strains used to investigate the correlation between wing damage and aggression were derived from a strain that was previously selected for increased aggression (Dierick and Greenspan 2006). Recombinants between a third chromosome derivative strain with high aggression were generated against a low-aggression wild-type Canton-S strain, and single-chromosome strains for the third chromosome were derived. These strains showed a range of aggressive behaviors and remained phenotypically stable throughout a 2-year period, when they were analyzed for aggressive behavior repeatedly and by independent investigators (data not shown). The isogenic SD1 line was created by crossing a single Canton-S male to multiple C(1)DX,*y w f* /Y females. To limit the mutation load per chromosome so that mutants would be healthy enough to show strong aggression phenotypes, the SD1 males were mutagenized with 10 mM ethyl methanesulfonate (EMS) using a similar feeding protocol as previously described (Haelterman *et al.* 2014). Each resulting male offspring was backcrossed to C(1)DX,*y w f* /Y females. All flies were reared on yeast, cornmeal, molasses, and agar food at room temperature (22.5 ± 0.5°) on a 16-hr light/8-hr dark cycle.

### Aggression assay

All aggression assays were performed in the arena assay as previously described (Dierick 2007) with minor modifications. On the day of eclosion, males were collected and group-housed for 4 days and then isolated for 2.5 days in individual 5 ml tubes with 0.4 ml of standard fly food. A minimum of 70 pairs of flies per genotype were tested and multiple genotypes were assessed simultaneously. Flies were recorded for 20 min and scored for aggression by observing unambiguous fighting behaviors of repeated lunging or boxing. All data are reported as average fighting frequencies per genotype plus SEM. For some experiments, pairs of flies from the same experiments were also analyzed using automated lunge counting analysis with the CADABRA software (Dankert *et al.* 2009). All data were reported as boxplots of lunge number in 20 min showing medians, 25th, and 75th percentiles (first and third quartiles), and whiskers indicating fifth and 95th percentiles.

### Wing damage assessment

On the day of eclosion, 15 males were collected and group-housed in a standard fly vial with standard fly food. Any fly with damaged wings at this point was discarded. The flies were transferred to new vials as necessary. After 7, 14, or 21 days, the flies were anesthetized, and wings were observed for any missing piece of the wing edge or wing blade. Each wing was scored independently. For imaging, the wings were removed at the proximal end with dissection scissors and were dry mounted and imaged via bright field microscopy on a Zeiss Axioplan2 (Zeiss [Carl Zeiss], Thornwood, NY).

### Flight assays

The flight assay was constructed as previously reported (Babcock and Ganetzky 2014). A total of 35–50 flies per group was tested to reach > 90% power determined by G-power. The data are reported as boxplots of the landing heights representing the medians and quartiles as the boxes with 5th and 95th percentiles as whiskers. In addition, a novel flight-choice assay was developed with the following components that allowed flies to choose to fly over a water moat. An inverted funnel, 65-mm wide and 70 mm in height, was glued to an inverted petri dish, 37 mm in diameter with a 12-mm opening in the center connecting the funnel to the dish. The inverted small petri dish platform was centered in a large petri dish to create a 52-mm wide moat of water surrounding the small petri dish platform. A clear plastic rectangular box measuring 165 mm in width and length, and 102 mm in height, was built on top of the large petri dish. Ten flies were introduced in the bottom of the inverted funnel through a 4-mm hole by gentle aspiration. A minimum of 200 flies was tested for each genotype/condition. The percentage of flies that successfully crossed the moat without touching the water was scored in each experiment. The data are presented as bar graphs of the average percentage of successful crossers plus SEM.

### Copulation assay

A 100-mm diameter petri dish with damp Whatman paper on the bottom was used for the copulation arena. Ten males and 10 females were loaded into the chamber and recorded for 1 hr. Time to initiate copulation and duration of copulation were recorded. Only pairs that started mating before the first pair to finish mating were included for analysis. For all assays, 3–5-day-old virgin Canton-S females were used and a total of 25–30 males were tested for all conditions. For the artificial wing damage, 3–4-day-old Canton-S males were lightly anesthetized with CO_2_ and their wings were cut with dissection scissors. They were allowed to recover from anesthesia for 24 hr before testing. For the naturally damaged wings, AI31d males were group-housed with 15 males per vial for 20 days, at which point the males were sorted for wing damage under CO_2_ anesthesia. The flies were allowed to recover for 24 hr before testing. All data are presented as boxplots that represent the latencies to mate and mating durations in minutes, showing the spread of the medians and first and third quartiles as boxes and the 5th and 95th percentiles as whiskers.

### Genome sequencing

Genomic DNA was isolated from SD1 males and all five mutant strains using the DNeasy Blood and Tissue kit according to the manufacturers protocol (QIAGEN, Valencia, CA). Whole-genome sequencing was performed by Genewiz (South Plainfield, NJ) at ∼20× coverage. Variants in the mutant strains were filtered by removing common SD1 variants, synonymous variants, noncoding variants, and variants with < 80% read counts.

### Statistical analysis

Aggression data are typically not normally distributed, and for these data medians were statistically compared using the nonparametric Kruskal–Wallis ANOVA for unpaired groups. We used the Mann–Whitney *U*-test for *post hoc* comparisons to identify those groups that differed to a statistically significant extent with Bonferroni correction for multiple comparison testing. Replicate numbers were determined to reach power > 80% using G-power. Correlation analysis between fighting frequencies and lunge number averages, and lunge number averages and average percent damaged wings, were performed using Pearson’s correlation analysis and *R*^2^ values, and *P*-values were determined. Flight and copulation data were analyzed by Kruskal–Wallis ANOVA. Statistically significantly different groups were identified using Mann–Whitney *U*-tests and Bonferroni correction. Wing-damage data were analyzed by ANOVA followed by Tukey–Kramer HSD (Honest Significant Difference) *post hoc* tests to identify statistically significantly different groups. Behavioral data are presented as bar graphs representing the mean percentages with SEM, or as boxplots representing the number of lunges, times to flight/copulation, or duration of copulation as medians, first and third quartiles as boxes, and 5th and 95th percentiles as whiskers to better visualize the spread of the data.

### Data availability

Whole-genome sequencing data have been deposited in the Sequence Read Archive (accession number PRJNA416149). Reagents published in this manuscript will be made available upon request.

## Results

### Aggressive behavior promotes wing damage

**Figure 1:**
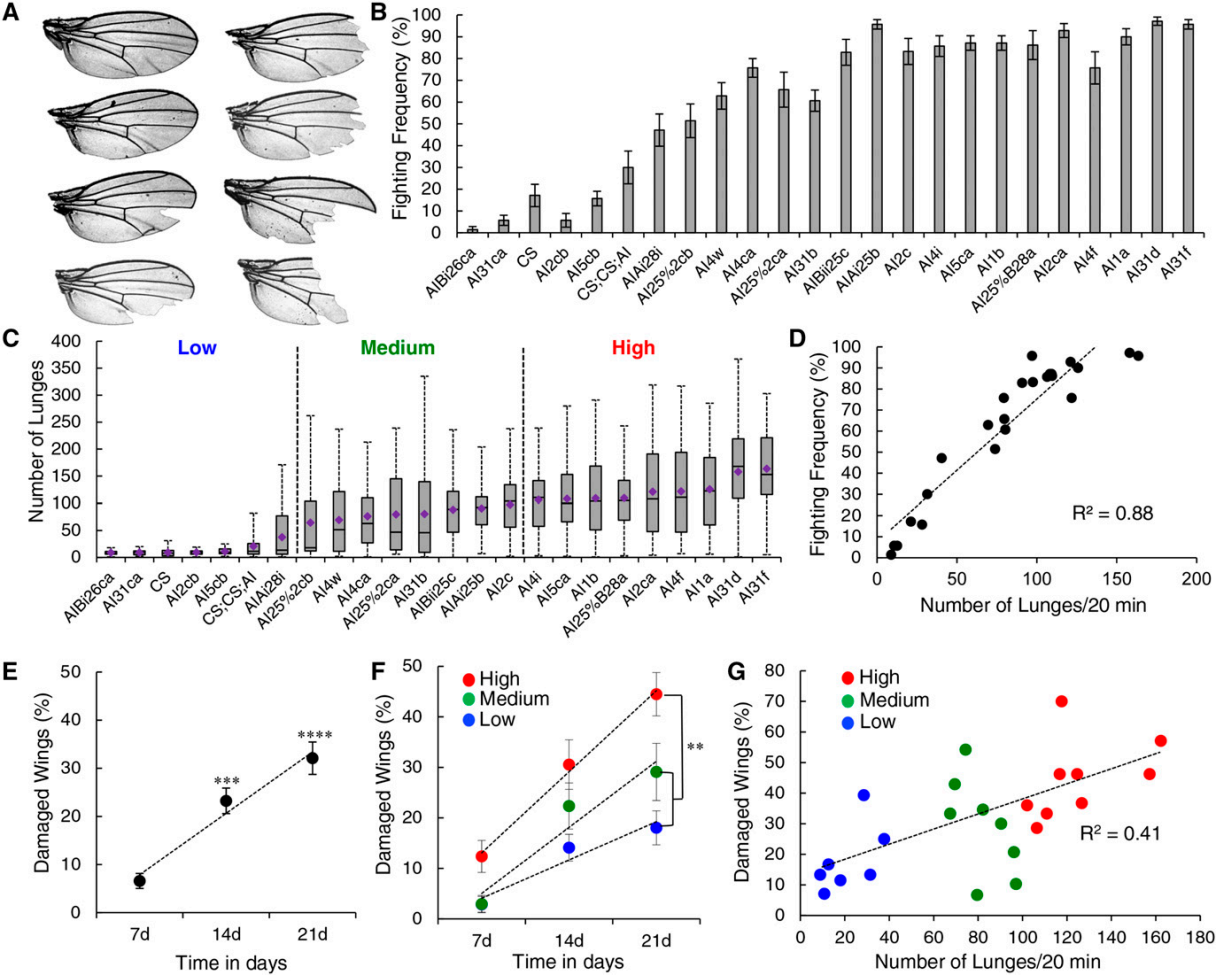
Wing damage correlates with aggression. (A) Example wings from group-housed aggressive males. Damage ranges from small nicks to missing the entire distal portion. (B) Fighting frequencies of 24 genetically related strains, ranging from low to high aggression, *n* = 70 pairs per genotype. (C) Lunge counts from CADABRA video analysis for the same strains as in (B) ranked in order of increasing mean lunge number. Strains were categorized into low- (< 40 lunges), medium- (40–100 lunges), and high- (> 100 lunges) aggression groups. *n* = minimum of 40 pairs per genotype. (D) Correlation between the two measurements of aggression: fighting frequency and mean number of lunges (*R*^2^ = 0.88, *P* < 0.0001). (E) The percentage of wings with damage increased over time across all strains. Both 14- and 21-day group-housed males had a significant increase compared to 7-day group-housed males (ANOVA, 21 day *vs.* 7 day: *P* = 0.0001; 14 day *vs.* 7 day: *P* = 0.0017; and 21 day *vs.* 14 day: *P* = 0.07). (F) The highly aggressive group of flies (red) had increased wing damage at all time points compared to the medium- or low-aggressive groups. At 21 days, the highly aggressive group had statistically significantly more wing damage than the medium- (green) and low-aggressive groups (blue) (two-way ANOVA, *P* < 0.01). (G) Wing damage and average number of lunges are positively correlated at 21 days (*R*^2^ = 0.41, *P* < 0.001). See also Figure S1 in File S1, and File S2.

In a range of animal species, aggressive encounters can lead to physical damage usually caused by biting, scratching, or clawing between fighting animals. *Drosophila* males do not have claws, teeth, or other weaponry and obvious damage to flies has not been documented. However, during fighting events, aggressive flies sometimes grab one or both wings of their opponent to destabilize or pull the opponent (Hoffmann 1987; Chen *et al.* 2002; Dierick and Greenspan 2006) (File S2). Such repeated holding or grabbing may eventually cause damage to the flies’ wings. Flies also sometimes tumble over the surface on which they fight. In addition, during escalated encounters, flies stand vertically on their hind legs to reciprocally box and tussle, and their wings brush up against the surface. We anecdotally observed in highly aggressive strains that group-housed males incur damage to the edge of the wing blade. Some wings had minor nicks to the wing edge while others had multiple notches in the wing, and some were even missing the entire distal half of the wing (Figure 1A). To thoroughly investigate whether there is indeed a correlation between wing damage and aggressive behavior, we examined wing damage in 24 genetically related fly lines with varying levels of aggression, as determined by our previously described arena assay (Dierick 2007). The lines showed a gradient of aggressive behavior from very few fighting pairs to nearly every pair fighting throughout the 20-min observation period (Figure 1B). We further quantified the levels of aggression in these strains by analyzing the lunge number counts using automated CADABRA software (Dankert *et al.* 2009) (Figure 1C) and found that both variables were highly correlated (Figure 1D, *R*^2^ = 0.88, *P* < 0.0001).

To assess wing damage for each strain, 15 males were group-housed in a standard food vial for 7, 14, and 21 days, at which point the wings were collected. Any wing with a broken edge was scored as damaged. Across all strains, the percentage of damaged wings significantly increased over time (Figure 1E, one-way ANOVA: 7 day *vs.* 14 day, *P* = 0.0017; 7 day *vs.* 21 day, *P* = 0.0001; and 14 day *vs.* 21 day, *P* = 0.07). To better correlate wing damage with aggression, we used the average lunge number as a more precise quantitative measure for aggression to group the strains into low- (with lunge numbers below 40 in the 20-min observation period), medium- (40–100 lunges) and high-aggressive (> 100 lunges) strains (Figure 1C). When we plotted the damage per time point in these three categories, we found significantly more damage per time point for the high-aggression strains compared to the medium- and low-aggression strains (Figure 1F, two-way ANOVA, groups: *P* = 4.3e^−6^, time: *P* = 3.5e^−10^, interaction: *P* = 0.3). The greatest difference occurred at 21 days, and thus this time point was chosen for a strain-by-strain correlation analysis.

**Figure 2:**
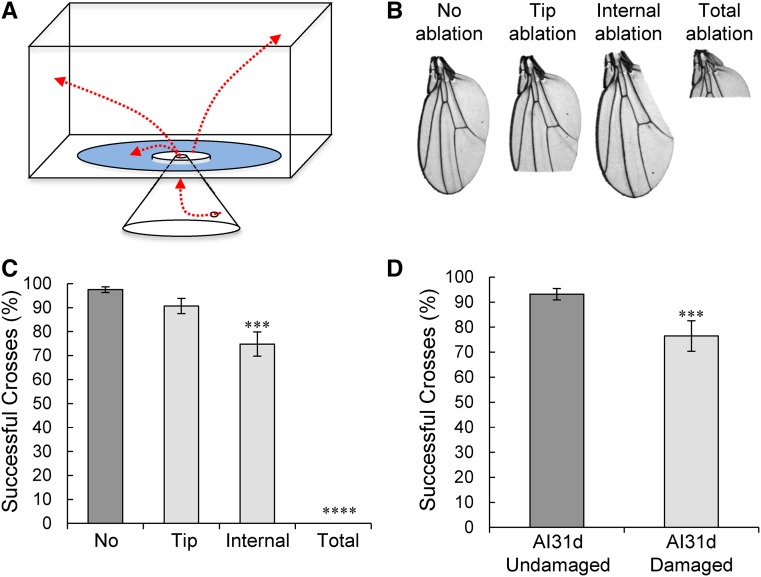
Wing damage impairs flight ability. (A) Schematic of the flight-choice assay: 10 flies are gently introduced in the upside-down funnel, climb up to the platform, and fly or jump off the platform. Surrounding the central platform is a moat of water that can only be cleared if flies can fly. Red lines indicate potential trajectories of movement of the flies that have been introduced into the funnel. Successful crosses are recorded. (B) Pictures of artificially damaged wings are shown next to an undamaged wing. Three types of ablation are compared: tip, internal edge, and total ablations. (C) Manually induced damage to the wings of Canton-S males slightly reduces the percentage of successful crosses while all flies with totally ablated wings never successfully crossed the water, *n* = 10 groups of 10 flies (ANOVA, no *vs.* tip: *P* = 0.37; no *vs.* internal: *P* < 0.001; and no *vs.* total: *P* < 0.0001). (D) Flies from the high-aggression AI31d strain were group-housed for 21 days and separated into damaged and undamaged flies 1 day before the flight assay. Males with wing damage were significantly less successful at crossing the water moat. *n* = 12 groups of 10 flies per condition (Mann–Whitney *U*-test, *P* = 1.22e^−4^). See also Figure S2 in File S1.

The comparison of aggression, as measured by the lunge number average, and wing damage at 21 days shows a strong and highly significant positive correlation (Figure 1G, *R*^2^ = 0.41, *P* < 0.001). We also found a significant correlation when comparing wing damage and aggression as recorded by fighting frequencies (Figure S1A in File S1, *R*^2^ = 0.28, *P* = 0.011). If damage were indeed caused by aggressive encounters, females should show much less or no damage, because females fight dramatically less than males and never show physical interactions that involve wing-holding or grabbing (Nilsen *et al.* 2004; unpublished results H. A. Dierick). However, if the damage were due to some sensitivity to general behaviors such as grooming or social interactions other than aggression, then females should show a similar phenotype as males of the same genotype. We tested this prediction on females from three different strains corresponding to the low-, medium-, and high-aggression male groups. Unlike the males, none of the females showed dramatic increases in wing damage after being group-housed for 21 days, suggesting that damage is indeed caused by repeated aggressive encounters (Figure S1B in File S1, ANOVA, males: all groups different *P* < 0.005, females: not significant). We next tested whether artificially induced wing damage alters aggressive behavior. We used a low-aggression Canton-S strain and manually damaged their wings by removing specific portions from both wings (Figure 2A). Baseline levels of aggression were unaltered, suggesting that wing damage does not increase aggressive behavior (data not shown). Flies that were fed 5-HTP (5-hydroxy-tryptophan, the immediate and rate-limiting precursor to 5-HT, 5-hydroxy-tryptamine, serotonin) to increase aggression (Dierick and Greenspan 2007) also showed no behavioral difference between damaged and undamaged flies (Figure S1C in File S1, Kruskal–Wallis ANOVA, *P* = 0.62), suggesting that their capacity to fight remains intact after wing damage. Finally, artificial damage to the wings of males from a high-aggression strain also had no significant effect on their fighting frequencies (Figure S1D in File S1, Kruskal–Wallis ANOVA, *P* = 0.18). Together, these data show that increased aggression in *Drosophila* can lead to permanent damage, but that damage itself does not prevent flies from fighting or dramatically alter their aggression (up or down), as even completely wingless flies from a highly aggressive strain still have extremely high fighting and escalation frequencies.

### Wing damage negatively affects flight ability

We next questioned whether the damaged wings that appear to result from repeated aggressive encounters negatively impact the fly and first assessed their flight capabilities. We used a previously reported flight assay (Babcock and Ganetzky 2014) to measure flight ability in males with or without manually induced wing damage. In this assay, flies are ejected into a flight cylinder with a sticky wall and their landing height is recorded (Figure S2A in File S1). A lower landing height suggests a defect in flight performance. Only the totally wing-ablated group had a significant difference in landing height compared to the undamaged flies (Figure S2B in File S1, ANOVA, *P* = 2.13e^−13^). Flies with curly wings also had impaired performance but flies with serrated wings did not (data not shown). Because naturally occurring wing damage is usually less severe than total ablation, this flight assay is likely not sensitive enough to measure potential changes in flight ability.

We wondered whether a flight defect might be more easily detected when flies have to initiate flight from a level surface. To examine this possibility, we designed a novel flight-choice assay. In this assay, flies are introduced into a small inverted funnel where they must climb up into the flight chamber (Figure 2A). Once they climb out of the funnel, they reach a small platform surrounded by a large moat filled with water. Flies loaded into the chamber almost immediately leave the platform by attempting to fly or jump across the water moat. However, the length of the water barrier is greater than the distance wingless flies can jump (Zumstein *et al.* 2004). We recorded the percentage of flies that successfully crossed without touching the water. Almost every fly with undamaged wings successfully crossed the moat without touching the water (Figure 2C). Flies with tip ablations showed a slight decrease in successful crosses (Mann–Whitney *U*-test, *P* = 0.37), and flies with internal edge ablations showed a further and significant reduction in the percentage of successful crosses (Figure 2C, Mann–Whitney *U*-test, *P* < 0.001). Flies with fully ablated wings never successfully crossed the moat without touching the water. These data suggest that the flight-choice assay is sensitive enough to pick up subtle forms of wing damage. We next tested flies with natural aggression-induced wing damage at 21 days of age after group-housing one of the high-aggression strains and found that males with damage were significantly less successful at crossing the water moat compared to undamaged siblings (Figure 2D, Mann–Whitney-*U*-test, *P* = 1.22e^−4^). We observed a similar but weaker effect in damaged compared to undamaged flies from group-housed medium aggression AI4w and low-aggression Canton-S strains (Figure S2, C and D in File S1, Mann–Whitney *U*-tests, *P* = 0.023 and *P* = 0.048, respectively). These results show that wing damage incurred after group-housing impairs the ability of damaged males to fly compared to their undamaged siblings, and that effect is larger in more aggressive strains.

### Wing damage negatively affects mating ability

**Figure 3:**
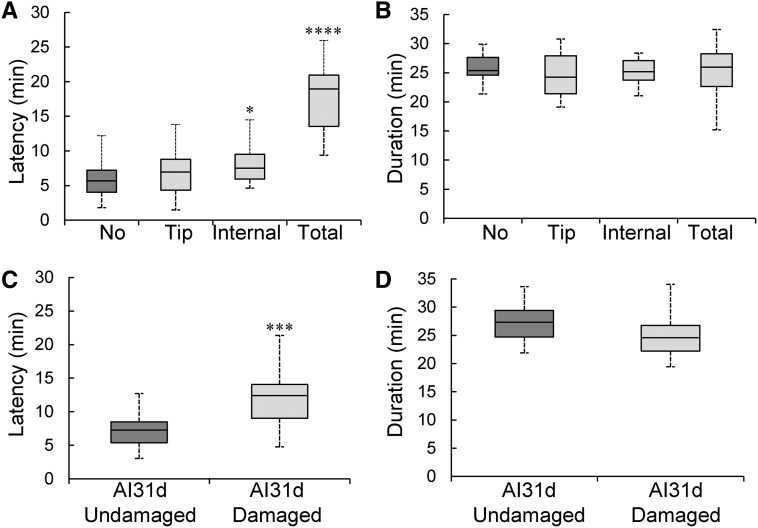
Wing damage increases copulation latency. (A) Canton-S males with manually damaged wings showed a small but increased latency to initiate copulation with a female, *n* = minimum of 25 males (Kruskal–Wallis ANOVA *P* = 1.25e^−10^; no *vs.* internal: *P* = 0.0026; and no *vs.* total: *P* = 1.3e^−8^). (B) However, the duration of mating did not significantly differ between males with different manually induced wing damage and controls (Kruskal–Wallis ANOVA, *P* = 0.63). (C) Naturally damaged wings also increase the latency to initiate copulation. The aggressive AI31d males were group-housed for 21 days before separating them into damaged and undamaged groups, *n* = minimum of 25 males (Mann–Whitney U, *P* = 5.3e^−5^, see also Figure S3, A and B in File S1). (D) Males with naturally damaged wings did not show significantly different mating durations (Mann–Whitney *U*-test, *P* = 0.7). See also Figure S3 in File S1.

Male flies not only use their wings to fly, but also to generate a courtship song to induce females to become receptive to mating (von Schilcher 1976; Greenspan and Ferveur 2000). If song production is impaired by wing damage, we predicted that this might lead to increased latencies to copulate. If wing damage would mostly affect the balance of the males as they copulate, we expected that the duration of copulation might be affected. Therefore, we tested whether wing damage altered the latency to successfully copulate and/or the duration of copulation. For the Canton-S males in which the wings were manually damaged through the ablation of specific regions (Figure 2B), the latency to mate significantly increased with internal and total wing ablation as compared to the flies with intact wings (Figure 3A, Kruskal–Wallis ANOVA, *P* = 1.26e^−10^; Mann–Whitney *U*-test no damage *vs.* internal: *P* = 0.0026; and no damage *vs.* total ablation: *P* = 1.19e^−8^). However, the duration of mating remained unchanged between these groups suggesting that males with damage are not otherwise impaired in mating (Figure 3B, Kruskal–Wallis ANOVA, *P* = 0.63). We next tested whether naturally acquired wing damage alters the time to mate. Indeed, highly aggressive males with wing damage at 21 days old showed a significant increase in latency to copulate as compared to their age-matched control males that had no visible damage (Figure 3C, Mann–Whitney U, *P* = 5.3e^−5^), while the duration of copulation was not significantly different between the damaged and undamaged flies (Figure 3D, Mann–Whitney U, *P* = 0.07). We obtained similar results with damaged *vs.* undamaged low-aggression Canton-S males and medium-aggression AI4W males (Figure S3, A and B in File S1). Altogether, these data suggest a negative fitness consequence for flies as a result of physically damaged wings incurred through fighting.

### Wing damage as a proxy to screen for aggression

**Figure 4:**
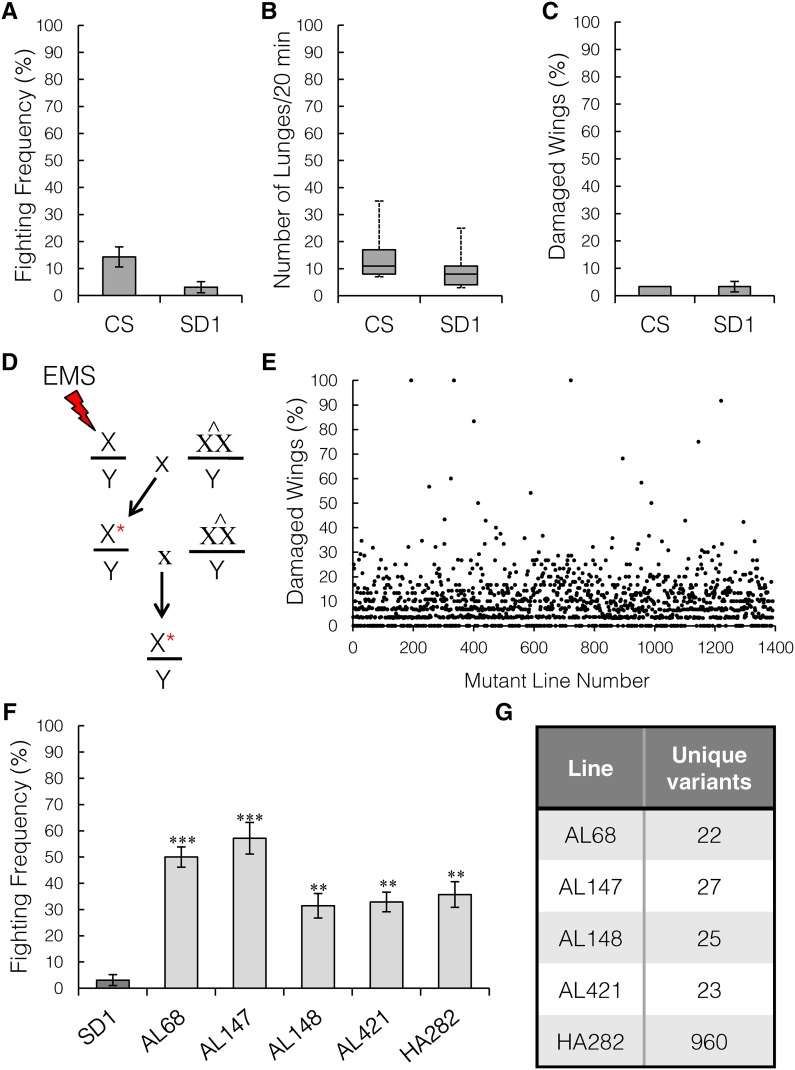
A wing-damage screen identifies aggression mutants. (A) The isogenic X chromosome line, SD1, shows low aggression like its parental Canton-S line (CS) as measured by fighting frequency, *n* = 70 pairs per genotype. (B) The same strains as in (A) also have low aggression based on the number of lunges, *n* = minimum of 55 pairs per genotype. (C) Wing damage at 21 days in group-housed SD1 and CS males is low, *n* = 45 males. (D) Schematic of the crosses for EMS mutagenesis. Red asterisks indicate mutant X chromosome. (E) A total of 1391 lines were screened for wing damage after 21 days of group-housing. The average across all lines was 9.7% damaged wings, and only 41 lines showed an increase of 30% or more. (F) Of the 41 lines with increased wing damage, five showed a significant increase in fighting frequency compared to the parental SD1 line, *n* = 70 pairs per genotype (Kruskal–Wallis ANOVA, *P* = 1.35e^−6^; Mann–Whitney *U*-test SD1 *vs.* AL68 or AL147, *P* < 0.002; and SD1 *vs.* AL148, AL421, or HA282, *P* < 0.005). (G) Filtering the variants from whole-genome sequencing from each strain revealed a low number of nonsynonymous, unique coding variants per line, except for HA282.

Because wing damage correlates well with aggression and is easy to score, we used this wing damage phenotype to perform the first EMS-induced, X chromosome, forward genetic screen to attempt to isolate mutants with increased aggressive behavior. Based on the results of the correlation analysis between wing damage and aggression, we predicted that mutant strains in which group-housed males have damage to > 30% of their wings by 21 days may also have increased aggression. We generated an isogenic X chromosome strain, SD1, from our standard laboratory Canton-S strain. Both strains showed very low aggression and minimal wing damage after 21 days of group-housing (Figure 4, A–C). To induce mutations, SD1 males were fed a low concentration of EMS to limit the mutation load per chromosome (Haelterman *et al.* 2014). All EMS-fed SD1 male flies were crossed to attached-XX/Y females (C(1)DX,*y w f*), and individual male progeny were crossed again to attached-XX/Y females to generate a stock from which multiple males with the same mutated X chromosome could be collected for group-housing (Figure 4D).

In total, 1391 independent mutant strains were tested for increased wing damage. Only 41 lines had > 30% damaged wings (Figure 4E). All 41 lines were tested for aggression and five lines had a significant increase as compared to the nonmutagenized parental SD1 line (Figure 4F, Kruskal–Wallis ANOVA, *P* = 1.35e^−6^; Mann–Whitney *U*-test SD1 *vs.* AL147: *P* = 0.002; and SD1 *vs.* AL148: *P* = 0.004). Of 60 control strains with < 30% damage, none had significantly increased aggressive behavior compared to the parental SD1 strain (data not shown). To identify the causal mutation, we performed whole-genome sequencing on all five mutants and the parental SD1 strain, and filtered the variants to find the unique, nonsynonymous coding mutations in each mutant strain. We decided to focus our further analysis on one of the top aggressive lines, AL68, which had 22 EMS-induced variants (Figure 4G, see also Table S1 in File S1).

### AL68 is a novel mutant in the *Shaker* locus

**Figure 5:**
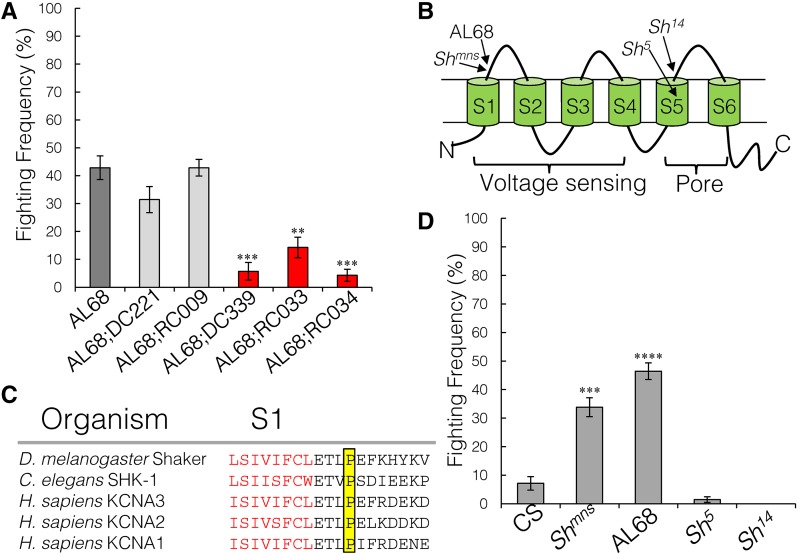
*Sh^AL68^* and *Sh^mn^*^s^ increase aggression and spontaneous hopping. (A) Genomic duplications covering *Hk* and *Shaker* were crossed into the AL68 background and assessed for aggression and hopping. Only the duplications covering *Shaker* suppressed aggression (red bars) and hopping, *n* = 70 pairs per genotype (Kruskal–Wallis ANOVA, *P* = 7.9e−8; Mann–Whitney *U*-test AL68 *vs.* AL68;DC339 *P* = 0.00024; and AL68 *vs.* AL68;RC033 *P* = 0.0009). (B) Schematic of Shaker, showing the six transmembrane domains. The arrows point to the locations of the independent point mutants. (C) Protein alignment of the S1 domain, in red, across different species. The proline that is substituted in AL68 is highlighted in yellow and is separated by one amino acid from the threonine mutation in *Sh^mns^*. (D) Fighting frequency of other mutations in *Shaker*. Of the previously isolated *Sh* alleles, only the *Sh^mns^* mutant had increased aggression compared to SD1, *n* = 70 pairs per genotype (Kruskal–Wallis ANOVA, *P* = 1.27e^−6^). See also Figure S4 in File S1. CS, Canton-S.

In addition to the increased aggression phenotype, AL68 also displayed a hyper-excitability or seizure-like phenotype. In isolation, the flies spontaneously hop and have short bouts of uncoordinated locomotor behavior. When placed with other flies of the same genotype, their hopping phenotype worsened, accompanied by longer bouts of uncoordinated movement. We found mutations in three strong candidates that might explain the seizure-like phenotype: *open rectifier potassium channel 1* (*Ork1*), *Shaker* (*Sh*), and *Hyperkinetic* (*Hk*). We predicted that rescuing the hyper-excitability phenotype would enhance the aggression phenotype because flies would be more coordinated. We used X chromosome duplication stocks to complement the mutations in *Hk* and *Sh* with a wild-type allele (Venken *et al.* 2010). Rescue constructs that cover the complete *Ork1* locus are not available. While duplications covering *Hk* did not rescue either phenotype in AL68, the duplications that cover the *Sh* locus rescued both the aggression (Figure 5A, Kruskal–Wallis ANOVA, *P* = 7.9e^−8^; Mann–Whitney *U*-test AL68 *vs.* AL68;DC339: *P* = 0.0002; and AL68 *vs.* AL68;RC033: *P* = 0.0009) and seizure-like phenotypes. The duplications that cover the remaining 19 unique variants in AL68 also did not rescue either phenotype (Figure S4 in File S1).

*Shaker* encodes the α-subunit of a tetrameric voltage-dependent potassium channel involved in membrane repolarization after action potentials. Each subunit consists of six transmembrane domains, S1–S6, where S1–S4 form the voltage-sensing region and S5–S6 form the pore region (Figure 5B). The mutation in AL68 is a C–T transition in exon 9, resulting in an evolutionarily invariant proline to serine substitution in the extracellular side of the S1 domain (Figure 5C). It is possible that this mutation alters the voltage-dependency of neuronal repolarization.

To confirm that mutations in *Shaker* alter aggressive behavior, we tested existing *Sh* alleles with other single-base pair mutations. A point mutation in *Sh^mns^* (Cirelli *et al.* 2005), which alters a threonine to isoleucine two residues N-terminal to the AL68 mutation, also showed increased aggression and a similar seizure-like phenotype to AL68 (Figure 5D, Kruskal–Wallis ANOVA, *P* = 1.27e^−6^). However, alleles with mutations in the pore domain, *Sh^5^* and *Sh^14^* (Lichtinghagen *et al.* 1990), did not have altered aggression or a seizing phenotype (Figure 5D).

## Discussion

Our work shows that males from strains with high levels of aggression develop damage to their wings when they are group-housed and that wing damage increases over time. We show that males with artificially induced or natural wing damage have impaired flight abilities and increased copulation latencies. Previous work has shown that a benefit of aggressive behavior is that males can father a greater proportion of offspring (Baxter *et al.* 2015), suggesting that this advantage is a drive to maintain the behavior in the species. Our work suggests that a trade-off exists between immediate mating success and later-life copulation latencies; even small impairments resulting from aggression may keep excessive aggressive behavior in flies at bay.

We used this easy-to-screen secondary phenotype of aggression to screen chemically mutagenized flies for wing damage to potentially identify highly aggressive mutants. From ∼1400 screened mutant X chromosomes, we found 41 mutants with excessive wing damage and five of these also showed increased aggression. While it is clear that not all wing damage is due to aggression, some of the strains with increased wing damage may have been caused by autosomal dominant mutations that caused increased aggression and may have been missed because the behavioral screen was performed several generations after the wing-damage screen, which would have led to the likely loss of these autosomal dominant alleles.

Using whole-genome sequencing and complementation mapping, we identified the causal locus in one of our mutants. This is a novel allele of the *Shaker* (*Sh*) locus, which encodes a voltage-gated potassium channel. Interestingly, our *Sh^AL68^* mutant also had a hyperexcitability phenotype making flies spontaneously hop and jump. Both phenotypes also occurred in *Sh^mns^*, which was isolated in a screen for short-sleeping flies and also affected the voltage-sensitive domain of the protein (Cirelli *et al.* 2005). While we did not examine sleep duration in our mutant, it is unlikely that sleep itself is responsible for the difference in aggression because sleep loss was recently shown to decrease rather than increase aggression in flies (Kayser *et al.* 2015). Two *Sh* alleles with mutations in the pore domain did not exhibit either phenotype. A possible explanation for this discrepancy is that these very severe mutations induce a homeostatic compensation mechanism, leading to upregulation of other K^+^-channels that compensate for loss of *Shaker* (Bergquist *et al.* 2010), thus suppressing both the aggression and seizure-like phenotypes. The mutations in *Sh^AL68^* and *Sh^mns^* may not be severe enough to induce a compensation mechanism. Regardless of such homeostatic compensation in different classes of *Sh* mutants, it is likely that increased excitability in different neuronal circuits may underlie both the short-sleeping phenotype and increased aggression. Further work will be needed to elucidate the precise mechanism that causes some *Sh* mutants to have increased aggression.

In summary, we have shown that flies incur physical damage to their wings, likely from repeated aggressive encounters. We used this phenotype to perform the first forward genetic mutagenesis screen and identified a novel allele in *Sh*, a gene that has so far not been implicated in aggression. How this mutation affects neuronal excitability and which neurons are affected to alter aggression remains unknown. Finally, the quick and easy screening method that we developed here can uncover the genetic components regulating the complex behavior of aggression.

## 

## Supplementary Material

Supplemental material is available online at www.genetics.org/lookup/suppl/doi:10.1534/genetics.117.300292/-/DC1.
