# Supplementary material for: Isolation of Aggressive Behavior Mutants in Drosophila Using a Screen for Wing Damage
Source: Genetics. 2017 Nov 6;208(1):273–82. doi: 10.1534/genetics.117.300292 (PMC5753862; doi:10.1534/genetics.117.300292)
Supplement: Supplementary file 1 [file 273FileS1.pdf]

Supplemental Material for:

Isolation of aggressive behavior mutants in *Drosophila* using a screen for wing damage

Shaun M. Davis[1], Amanda L. Thomas[1]†, Lingzhi Liu[1], Ian M. Campbell[1]‡, and Herman A. Dierick[1,2,3,4]

Department of Molecular & Human Genetics[1], Department of Neuroscience[2], Program in Developmental Biology[3], Baylor College of Medicine, Houston, TX 77030, USA

†Current address: Columbia University Medical Center, Laboratory of Genetics and Genomics, New York, NY 10027

‡Current address: The Children's Hospital of Philadelphia, University of Pennsylvania, School of Medicine, Department of Pediatrics, Philadelphia, PA 19104

[4]Correspondence should be addressed to Herman A. Dierick (Dierick@bcm.edu)

Table S1: Unique coding variants in AL68

Gene	Cytology	Position & mutation	Amino acid change	Genomic duplications
**east**	2C2-2B17	1909863: AC>-	NP_524864.2:p.[Thr437fs]; NP_001188530.1:p.[Thr437fs]; NP_001188531.1:p.[Thr437fs]; NP_001245484.1:p.[Thr437fs]; NP_001245485.1:p.[Thr437fs]	DC035, DC106, DC413
**rg**	4E2-4F2	5130913: G>A	NP_001138159.1:p.[Arg1522Gln]; NP_001138157.1:p.[Arg1739Gln]; NP_001036261.2:p.[Arg1729Gln]; NP_726978.1:p.[Arg1539Gln]; NP_001138158.1:p.[Arg1539Gln]	RC019
**CG34434**	5A8	5509458: C>A	NP_001096887.1:p.Val206Phe	DC140, DC141
**CG34434**	5A9	5509464: G>C	NP_001096887.1:p.Arg204Gly	DC140, DC141
**CG34434**	5A10	5509472: CC>GG	NP_001096887.1:p.Gly201Pro	DC140, DC141
**CG15765**	5C2-5C3	5732238: G>A	NP_572269.1:p.[Ala543Thr]; NP_001259263.1:p.[Ala543Thr]	DC144, DC145
**l(1)G0148**	6C3	6552197: T>C	NP_727103.1:p.Leu371Pro	DC026, DC160
**CG33223**	7F1	8359931: C>T	NP_996378.1:p.Glu139Lys	DC192
**CG15365**	8C4	8949925: TGT>-	NP_572516.1:p.Asn540_Ser541delinsSer	RC022, DC202
**CG34449**	8D7-8D9	9222294: CTGC>-	NP_001259382.1:p.[Gln600fs]; NP_727339.3:p.[Gln600fs]; NP_001096921.1:p.[Gln577fs]	DC206
**Hk**	9B5	10137832: GCGACT>-	NP_001259401.1:p.[Gln24_Pro26delinsPro]; NP_001259400.1:p.[Gln24_Pro26delinsPro]; NP_001259399.1:p.[Gln24_Pro26delinsPro]; NP_511104.3:p.[Gln24_Pro26delinsPro]	RC009, DC221
**CG17841**	9B12-9B14	10269172: C>G	NP_001096940.2:p.[Gly12Ala]; NP_727413.1:p.[Gly12Ala]	DC223
**CG17841**	9B12-9B14	10269172: C>G	NP_001096940.2:p.[Gly12Ala]; NP_727413.1:p.[Gly12Ala]	DC223
**Ork1**	9F7-9F8	10775645: T>C	NP_727466.1:p.[Ser270Gly]; NP_511112.1:p.[Ser270Gly]	
**CG15740**	10F1	11737758: C>A	NP_572753.2:p.Gln718His	DC245, DC246
**CG15740**	10F1	11738705: ->GCTGTG	NP_572753.2:p.Gln403delinsProGlnGln	DC245, DC246
**CG4004**	11B14	12640412: ACCACGC>-	NP_001259492.1:p.Asp3fs	DC258, DC259
**CG9411**	12E10	14368345: GGT>-	NP_572962.1:p.Pro489_Pro490delinsPro	DC284, DC285, RC030
**CG15646**	13E3-13E5	15562424: C>T	NP_573051.1:p.Glu302Lys	DC303
**Socs16D**	16D4	17719836: G>A	NP_523390.3:p.[Ser18Leu]; NP_001259662.1:p.[Ser18Leu]	DC335, DC336
**Sh**	16F3-16F6	17845468: G>A	NP_523393.3:p.[Pro250Ser]; NP_728120.1:p.[Pro205Ser]; NP_728123.1:p.[Pro250Ser]; NP_728122.1:p.[Pro238Ser]; NP_001188668.1:p.[Pro238Ser]; NP_996498.2:p.[Pro238Ser]; NP_001245727.1:p.[Pro238Ser]; NP_001259665.1:p.[Pro250Ser]; NP_996497.1:p.[Pro250Ser]; NP_001245726.1:p.[Pro198Ser]; NP_728124.1:p.[Pro205Ser]; NP_001162788.1:p.[Pro205Ser]; NP_001259664.1:p.[Pro238Ser]	DC339, RC033, RC034
**Nup205**	18F4	19752566: G>A	NP_728308.1:p.[Asp1883Asn]; NP_608362.2:p.[Asp1860Asn]	DC368

List of all the variants in AL68 that change amino acid sequences in the target genes. The right most column lists the 3rd chromosome P(acman) duplications (VENKEN *et al.* 2010) that cover the genes with the variants. Note that the variant in *Sh* affects annotation of the proline residue differently dependent on the alternative splicing isoform of the protein.

Supplemental Figures and Movies

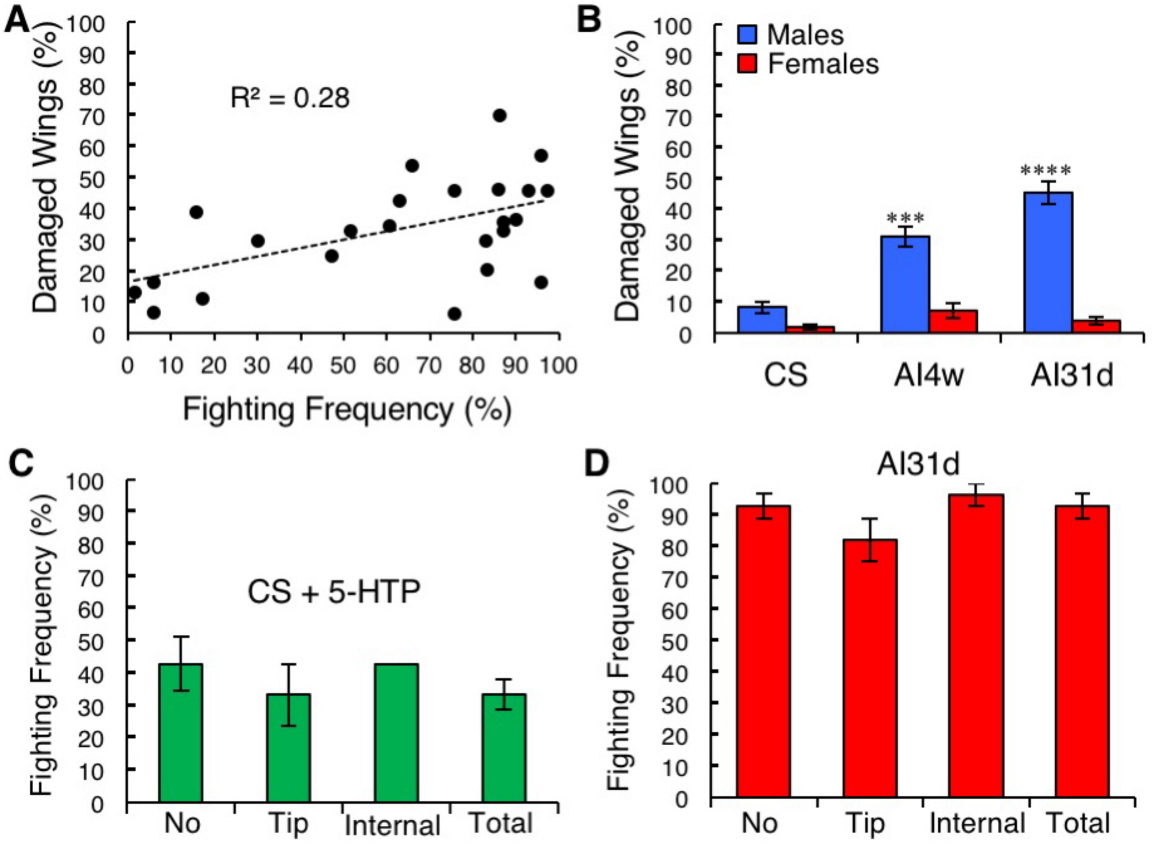

Figure S1: Wing damage correlates with aggression but does not alter aggression. (**A**) Wing damage and fighting frequency show a positive correlation (R^2=0.28, *P=0.011*). (**B**) Males from low (wild-type Canton S), medium (AI4w) and high (AI31d) aggressive strains have an increasing percentage of wing damage after being group housed for 21 days (ANOVA $P=3.39e^{-12}$, Tukey-Kramer HSD test, AI4w vs CS: *P=0.00011*, AI31d vs. AI4w: *P=0.0045*, AI31d vs. CS: *P=0.0001*). Females of the same strains, which show no aggression, have very little damage (ANOVA, Tukey-Kramer HSD test, AI4w vs CS: *P=0.94*, AI31d vs. AI4w: *P=0.99*, AI31d vs. CS: *P=0.7*). (**C**) Wild-type Canton S males fed 5-HTP for 4 d show fighting frequencies of ~40%. Artificial damage by ablating the tip, internal wing edge or the entire wing (Fig 2B) do not significantly alter fighting frequencies (Kruskal-Wallis ANOVA, *P=0.62*). (**D**) A very high aggression strain similarly shows no significant change in fighting frequency when wings are artificially damaged (Kruskal-Wallis ANOVA, *P=0.18*).

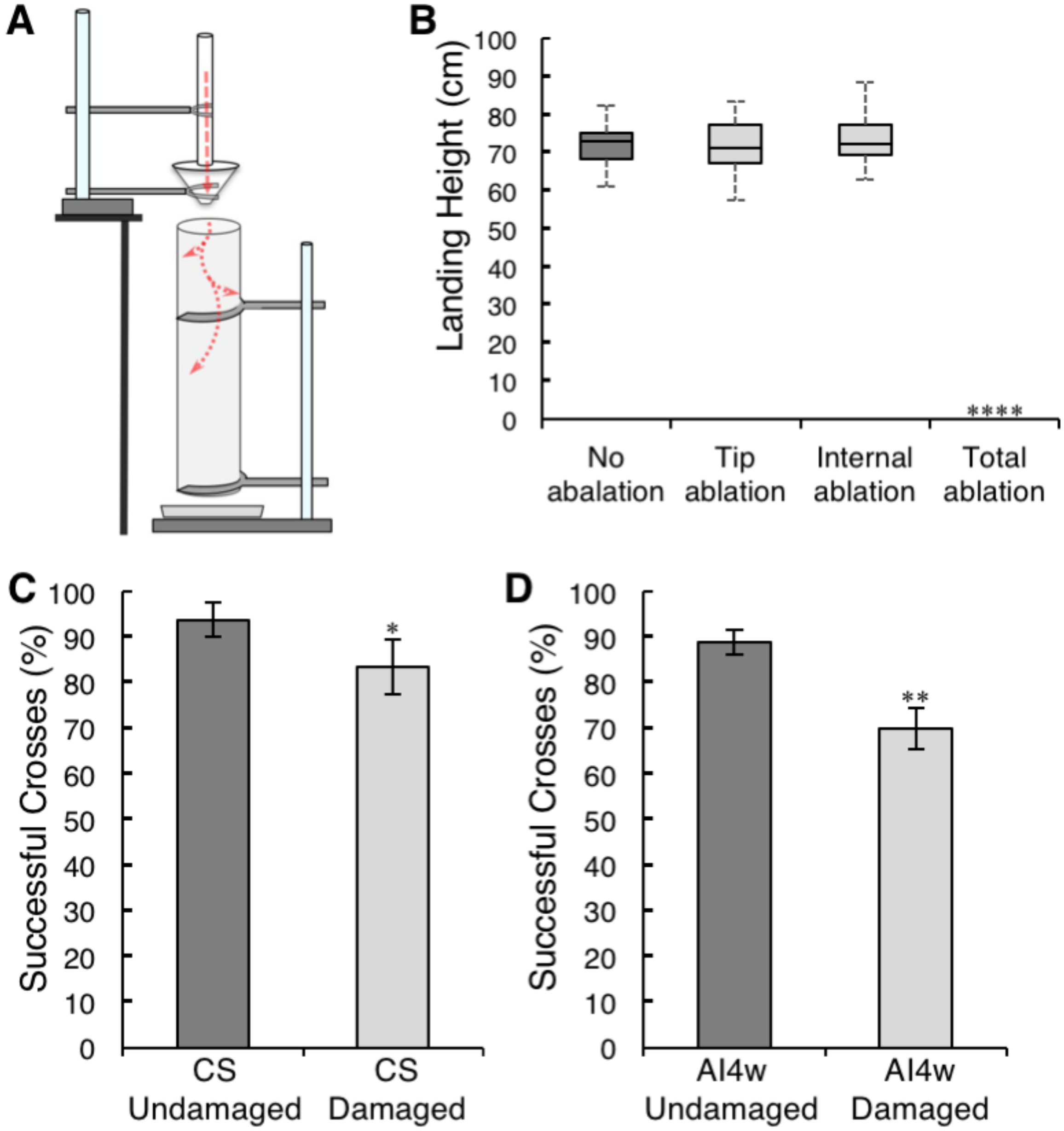

Figure S2: Wing damage impairs flight ability. (**A**) Schematic of the flight assay described by (BABCOCK AND GANETZKY 2014), up to 100 flies are launched through a loading tube into a cylinder that is coated with a sticky solution to evaluate flight ability by measuring landing height (higher landing heights correspond to better flight ability). (**B**) Three replicates of 30-50 Canton S males without damage or artificially induced wing damage (Fig 2B) were tested and landing heights recorded. Only males with totally ablated wings have significantly lower flight ability (Kruskal-Wallis ANOVA, $P=2.13e^{-18}$). Females have indistinguishable landing height distributions from males (data not shown). Flies with curly and dumpy wings also have reduced flight ability, while serrated

flies do not (data not shown). (**C**) Flies from the low aggression Canton S strain were group-housed for 21d and separated into damaged and undamaged flies 1 day before the flight assay. Males with wing damage were significantly less successful at crossing the water moat. n = 10 groups of 10 flies per condition (Mann-Whitney U-test, $P=0.048$). (**D**) Flies from the medium aggression AI4w strain were group-housed for 21d and separated into damaged and undamaged flies 1 day before the flight assay. Males with wing damage were significantly less successful at crossing the water moat. n = 10 groups of 10 flies per condition (Mann-Whitney U-test, $P=0.023$).

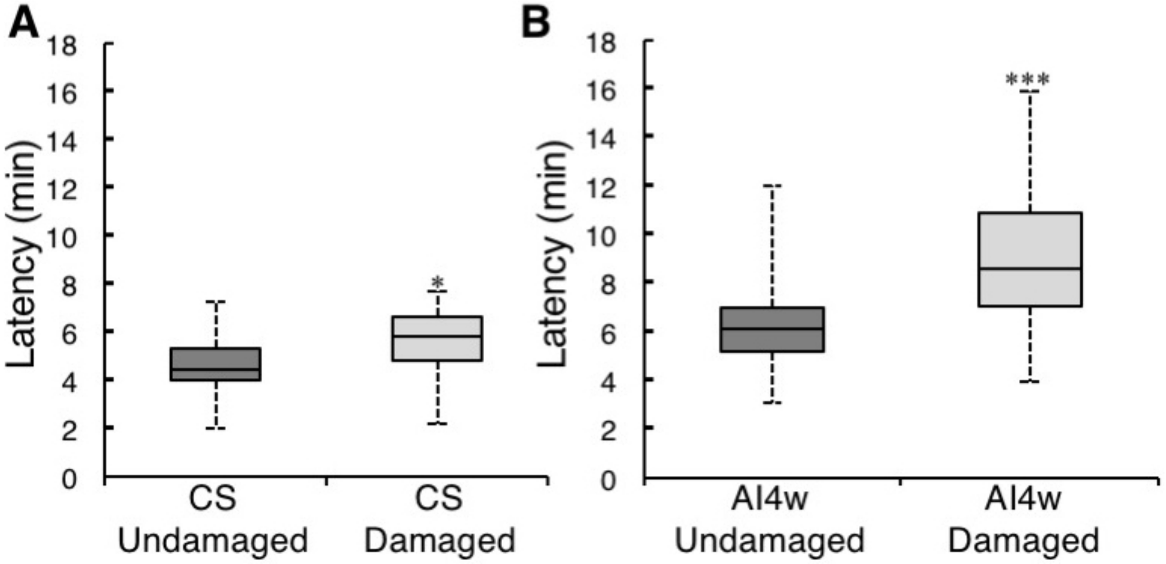

Figure S3: Aggression-induced damage increases copulation latency. (**A**) Wild-type Canton S males have low levels of aggression but still have damage to a percentage of their wings after being group-housed for 21 days. Wing-damaged males have slightly increased copulation latencies compared to the undamaged co-housed males (Mann-Whitney U Test, *P=0.005*). (**B**) Males with wing damage from a medium high aggression strain, AI4w, have significantly increased copulation latencies compared to the undamaged co-housed males (Mann-Whitney U Test, $P=04.95e^{-4}$).

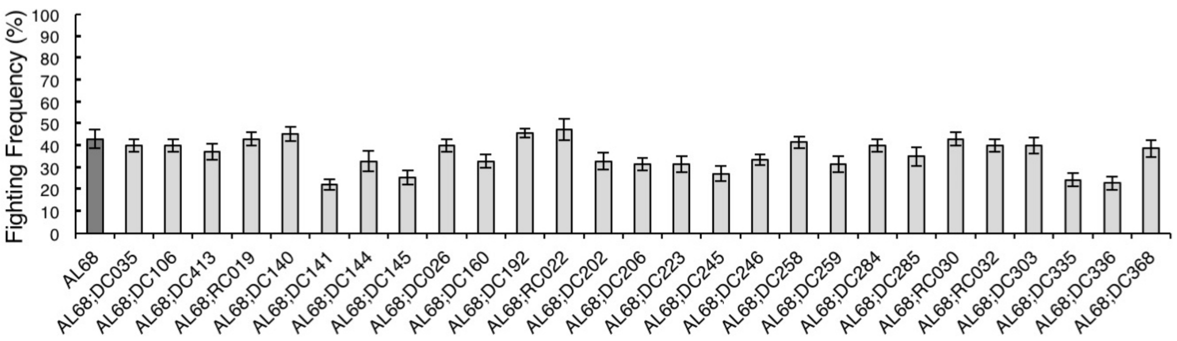

Figure S4: Complementation of other variants in A68 using P(acman) duplications (VENKEN *et al.* 2010). None of the duplications that cover the other unique variants in AL68 rescued the mutant phenotype.

Video S1: Fighting flies sometimes grab onto their opponent's wings. First scene: one fly grabs a single wing of his opponent during a hold (shown in slow motion). Second scene: one fly grabs both wings of his opponent during a hold (shown at normal speed). Repeated wing grabbing may cause damage to the wings over time.
